# The impact of body composition and fat distribution on blood pressure in young and middle-aged adults

**DOI:** 10.3389/fnut.2022.979042

**Published:** 2022-09-02

**Authors:** Song Zhao, Jiamin Tang, Yifan Zhao, Chong Xu, Yawei Xu, Shikai Yu, Yi Zhang

**Affiliations:** Department of Cardiology, Shanghai Tenth People's Hospital, Tongji University School of Medicine, Shanghai, China

**Keywords:** blood pressure, body composition, adipose tissue, trunk fat mass, leg fat mass

## Abstract

**Background:**

The relative contributions of each component of body composition to blood pressure (BP) remain unclear.

**Objective:**

We aimed to comprehensively investigate the impact of body composition and fat distribution on BP and quantify their relative contributions to BP in a large cohort with young and middle-aged adults.

**Methods:**

14,412 participants with available data on whole-body DXA measurement from the National Health and Nutrition Examination Survey were included. Multiple stepwise linear regressions of BP on components of body composition and fat distribution were built. Then, relative importance analysis was performed to quantify the contributions of each component to BP.

**Results:**

The median age of participants was 36 years and there were 50.7% women. Linear regression with mutual adjustment showed that total fat mass, total muscle mass, and trunk fat mass significantly and positively associated with BP; however, arm and leg fat mass significantly and negatively associated with BP. In men, after further adjusted for potential covariates, SBP were significantly determined by trunk fat mass (β = 0.33, *P* < 0.001), leg fat mass (β = − 0.12, *P* < 0.001), and total muscle mass (β = 0.10, *P* < 0.001); and DBP were significantly determined by trunk fat mass (β = 0.52, *P* < 0.001), leg fat mass (β = −0.15, *P* < 0.001), arm fat mass (β = −0.23, *P* < 0.001), and total muscle mass (β = 0.06, *P* < 0.001). Similar results were observed in women. Relative importance analysis showed that trunk fat mass was the major contributor (38–61%) to both SBP and DBP; meanwhile, total muscle mass also made relatively great contribution (35–43%) to SBP.

**Conclusion:**

Both fat mass and muscle mass independently associated with and substantially contributed to SBP in both men and women. After full adjustment, trunk fat mass positively associated with both SBP and DBP, and was the most dominant contributor to BP; however, leg fat mass negatively associated with both SBP and DBP.

## Introduction

Hypertension is one of the most important modifiable risk factors for cardiovascular diseases and also one of the leading causes of human deaths, with over 1.39 billion people suffering from hypertension and 10.4 million deaths per year in the world ascribed to hypertension (1, 2). The prevailing of hypertension is closely related to the global pandemic of overweight and obesity in the temporary society (3), as indicated that excessive weight gain accounts for 65–75% of the risk for primary hypertension (4). As a widely used surrogate for representing the nutrition status of individuals and the gold standard of defining overweight and obesity, body mass index (BMI) has been extensively studied and demonstrated to be significantly associated with blood pressure (BP), cardiometabolic risk, and mortality (5–7). However, BMI is not a perfect metric and has its own inherent flaws, especially when “BMI paradox” phenomenon was observed (8, 9). The most criticized facet of BMI is that it cannot reflect the body composition and the distribution of adipose tissue. Hence, the focus of investigation on overweight and obesity has been narrowed from general obesity to components of body composition and fat tissue in various depots, in order to better understand and evaluate the impact of extra weight gain on human cardiometabolic health.

Fat mass and lean/muscle mass are two important components of body composition. Quite a few studies have consistently demonstrated the firmly epidemiological link between total body fat mass and raised prevalence of hypertension or BP elevation (10–12). Moreover, fat tissue in various depots was indicated to have distinct impacts on BP. Trunk fat mass significantly associates with BP elevation or incident hypertension (13); however, accumulation of adiposity in limbs, especially in lower limbs, was found to be counterintuitively and negatively associated with hypertension or raised BP (14–17). In terms of lean/muscle mass, its effect on BP has not acquired as much attention and research interest as fat mass did, although weight gain is a combination of gain in both fat and lean mass (18, 19). In fact, epidemiological data have indicated that total lean/muscle mass was significantly and positively associated with BP, even independent of total fat mass (11, 20, 21). Nevertheless, it should be noted that components of body composition correlates closely to each other (22), and thus it is highly important to adjust for the other components when investigating the association of one component with raised BP or hypertension. The corresponding adjustment needs to consider two dimensions: the adjustments for adiposity in the other depots (for example, trunk fat mass vs. leg/arm fat mass); and the adjustment for the other component such as fat vs. lean/muscle mass. In addition, the relative contributions of various components of body composition and fat distribution on BP have not been reported by far.

Thus, in the present study, by constructing models simultaneously including body fat mass in various depots and total body muscle mass, we aimed to comprehensively investigate the impact of body composition and fat distribution on BP, based on a large cohort with young and middle-aged adults. Additionally, we determined the relative contributions of various components of body composition on BP variations for the first time.

## Methods

### Study population

This study was performed based on data from the National Health and Nutrition Examination Survey (NHANES), which is a nationally representative survey of the civilian and noninstitutionalized population of United States and conducted in two-year cycles since 1972. The detailed introduction and information of NHANES can be found on the website (https://wwwn.cdc.gov/nchs/nhanes/default.aspx). The present study was based on the analyses on the combined data of 8 cycles of NHANES (1999–2000, 2001–2002, 2003–2004, 2005–2006, 2011–2012, 2013–2014, 2015–2016, and 2017–2018) with whole-body dual-energy X-ray (DXA) measurements.

In total, there were 24,349 participants with data DXA data and aged between 18 and 59 years. We further excluded 422 participants without BP measurements or taking antihypertensive drugs, 370 participants with self-reported history of cardiovascular diseases including coronary artery disease, stroke, myocardial infarction, and/or heart failure, and 6,235 participants with missing data on covariates. Finally, a total of 14,412 participants were included for the present study.

### Anthropometry and measurements of body composition

Body weight, standing height, and waist circumference were directly measured by trained health technicians, according to a standard protocol. BMI was calculated as weight in kilograms divided by height in meters squared. The whole-body composition was acquired on the Hologic Discovery model A densitometers (Hologic, Inc., Bedford, Massachusetts, US), by trained and certified technologists, following the instructions provided by the manufacturer. Full and regional (trunk, legs, and arms) body measurements were automatically provided by the software. The original data of DXA body composition are described in details on NHANES website (https://wwwn.cdc.gov/nchs/nhanes/default.aspx). In the present study, we were mainly interested in fat mass in total body, trunk, leg, and arm region and total muscle mass. Besides, ratios of fat/muscle mass (FMR) in trunk, leg, and arm were calculated for further analysis. Ratio of trunk/leg fat mass was calculated and analyzed as well.

### Assessment of covariates

A detailed description of covariates is available on the NHANES website. Information on demography and lifestyle including sex, age, race/ethnicity, education attainment, smoking status, physical activity, and history of diseases were obtained by standard survey questionnaires. Race/ethnicity was categorized into non-Hispanic White, non-Hispanic Black, Mexican American, other Hispanic group, and other ethnic groups. Smoking status was grouped as never who reported smoking <100 cigarettes during their lifetime and no smoking now, ex-smoker who smoking >100 cigarettes during their lifetime and no smoking now, current smoker who smoking now. Regular moderate and regular vigorous physical activity were categorized as yes or no.

Trained physicians measured BP according to a standard protocol. BP was repeatedly measured three times and the average of the three measurements was used for analysis. Blood lipid profile including total cholesterol, triacylglycerol (TG), and high-density lipoprotein cholesterol (HDL-C) and blood glucose, glycated hemoglobin, were obtained according to a standardized protocol. Low-density lipoprotein cholesterol (LDL-C) were calculated using Friedewald formula (23). Dyslipidemia was defined as any of the following abnormalities: total cholesterol ≥ 200 mg/dL; LDL-C ≥ 130 mg/dL; TG ≥ 150 mg/dL; HDL-C ≤ 40 mg/dL in men and ≤ 50 mg/dL in women; or reported use of lipid-lowering medication. Diabetes mellitus was defined as history of diabetes or Glycated hemoglobin≥6.5%.

### Statistical analysis

Continuous variables were presented as mean ± SD for those with normal distribution or median (interquartile range) for those with skewed distribution; categorical variables were presented as absolute numbers and percentage in parenthesis. The differences in characteristics between sexes were detected using the two-sample student's *t*-test or Mann-Whitney *U*-test, whenever appropriate, for continuous variables, and the chi-square test for categorical variables. All analyses for the association between BP and body composition were performed separately for men and women because of the significant differences in body composition between sexes (as seen in Table 1 and Supplementary Figure S1). Analyses were done using SAS software, version 9.4 (SAS Institute, Cary, NC, USA) and R 4.1.2 (R Project for Statistical Computing, www.r-project.org). Statistical significance was defined as *P* < 0.05.

**Table 1:** Characteristics of the study participants.

**Variables**	**Total (*n* = 14,412)**	**Women (*n* = 7,099)**	**Men (*n* = 7,313)**	***P***
**Demographics**				
Age, year	36 (27, 46)	36 (27, 46)	35 (26, 45)	0.004
**Race/ethnicity, n (%)**				0.015
Mexican American	2,865 (19.88)	1,427 (20.1)	1,438 (9.66)	
Other Hispanic	1211 (8.4)	638 (8.99)	573 (7.84)	
Non-Hispanic White	5,735 (39.79)	2,842 (40.03)	2,893 (39.56)	
Non-Hispanic Black	2,729 (18.94)	1,306 (18.4)	1,423 (19.46)	
Other ethnic groups	1,872 (12.99)	886 (12.48)	986 (13.48)	
**Education level, n (%)**				<0.001
<12 years	3,090 (21.44)	1,414 (19.92)	1,676 (22.92)	
12–15 years	3,398 (23.58)	1,535 (21.48)	1,873 (25.61)	
>15 years	7,924 (54.98)	4,160 (58.6)	3,924 (51.47)	
**Smoking status, n (%)**				<0.001
Never	8,688 (60.28)	4,804 (67.67)	3,884 (53.11)	
Ex-smoker	2,305 (15.99)	944 (13.3)	1,361 (18.61)	
Current	3,419 (23.72)	1,351 (19.03)	2,068 (28.28)	
**Physical activity, n (%)**				<0.001
No	5,664 (39.3)	2,942 (41.44)	2,722 (37.22)	
Moderate	3,383 (23.47)	1,939 (27.31)	1,444 (19.75)	
Vigorous	5,365 (37.23)	2,218 (57.32)	3,147 (43.03)	
**Measurements of body composition and BP**
Body mass index, kg/m^2^	27.7 ± 6.2	28.0 ± 6.9	27.4 ± 5.4	0.134
Waist circumference, cm	93.7 ± 15.2	92.1 ± 15.7	95.4 ± 14.5	<0.001
Total fat mass, kg	23.9 (18.1 31.6)	27.0 (20.5, 35.4)	21.4 (16.1, 27.6)	<0.001
Total muscle mass, kg	50.6 ± 12.2	42.3 ± 8.0	58.7 ± 9.8	<0.001
Trunk fat mass, kg	11.4 (7.9, 15.6)	12.4 (8.6, 16.9)	10.6 (7.1, 14.2)	<0.001
Trunk muscle mass, kg	25.0 ± 5.8	21.4 ± 4.0	28.6 ± 4.9	<0.001
Leg fat mass, kg	8.5 (6.4, 11.3)	10.2 (8.0, 13.2)	7.0 (5.4, 9.1)	<0.001
Leg muscle mass, kg	16.4 ± 4.3	13.6 ± 3.1	19.0 ± 3.6	<0.001
Arm fat mass, kg	2.8 (2.1, 3.8)	3.3 (2.4, 4.4)	2.5 (1.9, 3.2)	<0.001
Arm muscle mass, kg	6.1 ± 2.1	4.4 ± 1.0	7.7 ± 1.5	<0.001
Whole-body FMR	0.53 ± 0.21	0.67 ± 0.18	0.38 ± 0.12	<0.001
Trunk FMR	0.50 ± 0.21	0.61 ± 0.21	0.39 ± 0.14	<0.001
Leg FMR	0.60 ± 0.26	0.80 ± 0.20	0.40 ± 0.13	<0.001
Arm FMR	0.57 ± 0.31	0.80 ± 0.26	0.34 ± 0.13	<0.001
TLR	1.35 ± 0.41	1.21 ± 0.37	1.48 ± 0.31	<0.001
SBP, mmHg	114 ± 14	115 ± 14	120 ± 13	<0.001
DBP, mmHg	71 ± 11	70 ± 10	72 ± 11	<0.001
**Blood lipid profile**				
Total Cholesterol, mg/dL	190.5 ± 38.7	189.4 ± 37.6	191.5 ± 39.7	<0.001
LDL-C, mg/dL	113.2 ± 34.5	110.2 ± 33.1	116.1 ± 35.5	<0.001
HDL-C, mg/dL	52.6 ± 14.9	56.9 ± 15.3	48.5 ± 13.1	<0.001
Triglycerides, mg/dL	103.0 (69.0, 156.0)	93.0 (65.0, 139.0)	114.0 (76.0, 174.0)	<0.001
**Comorbidities**, n (%)				
Diabetes mellitus	718 (4.98)	362 (5.1)	356 (4.87)	0.524
Anti-diabetes medication	370 (2.57)	190 (2.68)	180 (2.46)	0.414
Dyslipidemia	9,358 (64.93)	4,593 (64.70)	4,765 (65.16)	0.564
Lipid-lowering medication	418 (2.90)	184 (2.59)	234 (3.20)	0.030
Overweight	4,768 (33.08)	1,975 (27.82)	2,793 (38.19)	<0.001
Obesity	4,190 (29.07)	2,320 (32.68)	1,870 (25.57)	<0.001

Continuous variables are presented as means±standard deviation or median (interquartile range), and qualitative parameters as numbers with the percentage in parentheses.

FMR, fat mass to muscle mass ratio; TLR, trunk fat to leg fat ratio; SBP, systolic blood pressure; DBP, diastolic blood pressure; LDL-C, low-density lipoprotein cholesterol; HDL-C, High-density lipoprotein cholesterol.

The major purpose of this study is to investigate the influence of body composition and fat distribution on BP. The associations of BP with total body fat mass and trunk fat (central adipose) mass have been well established (10–13). Considering the close correlations between total body fat mass and lean/muscle mass and between trunk and leg fat mass as shown by other studies (22) and our data (Supplementary Figures S2, S3), it is highly important to adjusted total fat mass and trunk fat mass when investigating the associations of BP with total body muscle mass and leg fat mass. Thus, we conducted several exploratory analyses to determine the true associations of BP with total muscle mass and with leg fat mass, respectively. Firstly, we built a simple linear regression of total muscle mass on total fat mass and outputted the residuals, then continuingly built a linear regression of BP on the corresponding residuals to determine the fat-adjusted association of BP with total muscle mass. Same residual analysis was done for the association between leg fat mass and BP with adjustment for trunk fat mass. Secondly, we explored the interaction between total fat mass and total muscle mass in determining BP, by grouping participants into nine groups according to the tertiles of total fat mass and total muscle mass; then a simple linear regression model was constructed with SBP/DBP as the dependent variable and tertiles of total fat/muscle mass as the independent variable for analysis of the overall trend. The interaction between trunk fat mass and leg fat mass was also done by the same approach.

Furthermore, we constructed stepwise multiple linear regression of BP on various components of body composition and fat distribution including total muscle mass, trunk fat mass, leg fat mass, and arm fat mass, without (model 1) and with adjustment for covariates which were always included in the model (model 2 and 3). The corresponding covariates included in model 2 were age, race/ethnicity, education level, physical activity, diabetes mellitus, LDL-C, total cholesterol, anti-diabetes medication and lipid-lowering medication; and model 3 further included BMI on the basis of model 2. The significance levels for entry and for stay were set at 0.1. The multicollinearity between variables was determined by variation inflation factor>5. In all regression models constructed, no significant multicollinearity was observed. In sensitivity analysis, these regression analyses were performed in subgroups stratified by BMI classifications (normal weight, overweight, and obese) and in subgroups with and without dyslipidemia and/or diabetes.

Following the results of stepwise multiple regression, relative importance analysis was then performed to quantitatively evaluate the proportionate contributions of the corresponding components of body composition (variables remained in the regression model) to BP, using calc.relimp function in R language.

In addition, it has been indicated that fat mass represents metabolic load and muscle mass represents metabolic capacity, and the interaction between both determines the metabolic risk (24). The fat/muscle mass ratio, combined information on both metabolic load and capacity, was considered a useful marker. Therefore, we also organized stepwise multiple linear regression model to further confirmed the distinct impact of central and peripheral adiposity independent of muscle mass in determining BP, by using ratios of fat/muscle in trunk, leg and arm as independent variables and meanwhile adjusting for covariates including age, race/ethnicity, education level, physical activity, diabetes mellitus, LDL-C, total cholesterol, anti-diabetes medication, lipid-lowering medication, and BMI. Of note, all covariates were always included in the regression model.

## Results

### Characteristics of participants

Characteristics of the study participants are presented in Table 1. The median age of participants was 36 years and there were 7,099 (50.7%) women. Men and women were similar in BMI and prevalences of diabetes and dyslipidemia, but significantly different in other demographic, parameters of body composition and blood lipid profiles. In particular, men showed significantly higher waist circumference, muscle mass, trunk/leg fat ratio, SBP, and DBP, but lower fat mass and fat/muscle ratios than women. Significant correlations were widely found between components of body composition (see Supplementary Figure S2 for men and Supplementary Figure S3 for women).

### Influence of total fat and muscle mass on BP

**Figure 1:**
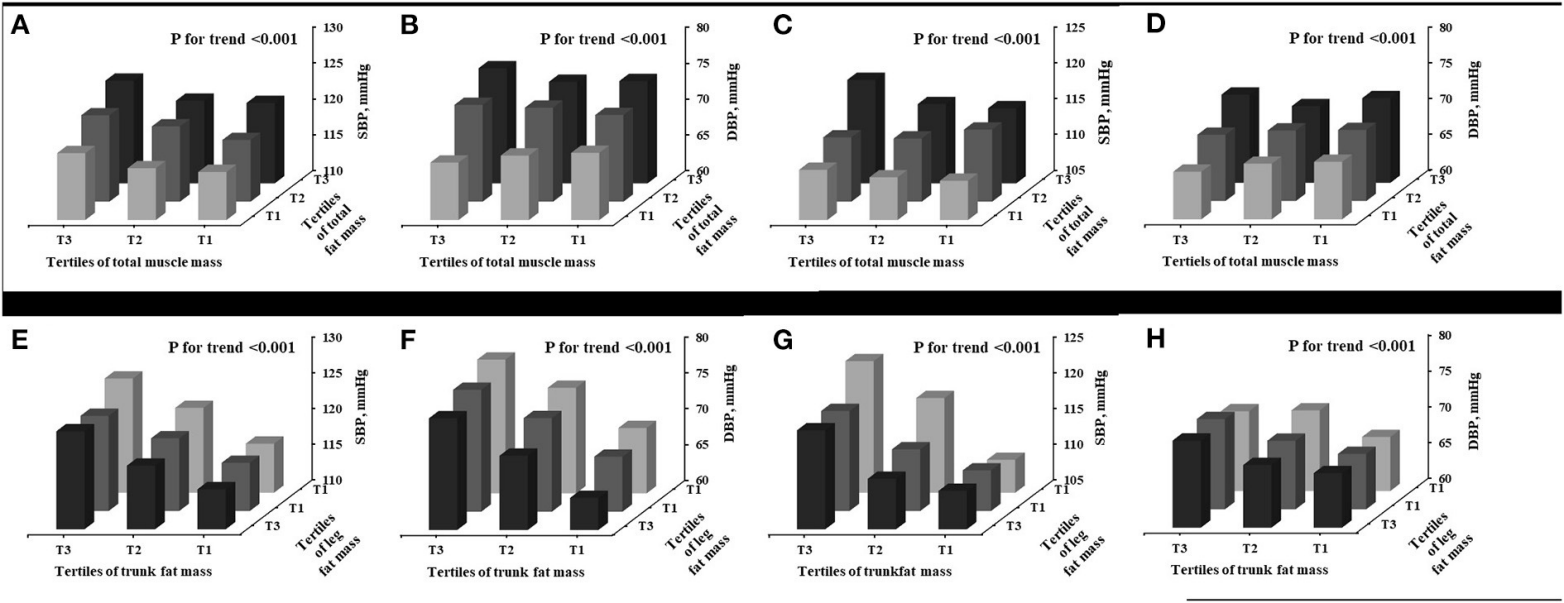
Interaction between various components of body composition in determining BP. Men **(A,B,E,F**); Women **(C,D,G,H)**. SBP, systolic blood pressure; DBP, diastolic blood pressure.

Men and women were separately grouped by tertile of total fat mass and total muscle mass, to investigate the interaction of both components' impact on SBP and DBP, respectively, as shown in Figures 1A–D. Overall, there was a graded increase from the lowest to the highest tertile of total fat and muscle mass for SBP and DBP in men and for SBP in women (*P* < 0.001 for trend for all tests). In the further residual analyses, after removing the influence of total fat mass, total muscle mass was positively associated with SBP and DBP in men and with SBP in women, but negatively associated with DBP in women; however, the statistical significance was only observed for SBP in men (β = 0.15, *P* < 0.001) and DBP in women (β = −0.076, *P* = 0.002), as shown in Supplementary Figure S4.

### Influence of trunk fat mass and leg fat mass on BP

As shown in Figures 1E–H, increasing trends in SBP and DBP were observed from the lowest to the highest tertile of trunk fat mass and from the highest to lowest tertile of leg fat mass for both men and women (*P* < 0.001 for trend for all tests). The further residual analyses showed that, after removal of the influence of trunk fat mass, leg fat mass was significantly and negatively associated with SBP and DBP in both men (β = −0.665, *P* < 0.001 for SBP; β = −1.219, *P* < 0.001) and women (β = −0.480, *P* < 0.001 for SBP; β = −0.264, *P* < 0.001 for DBP), as seen in Supplementary Figure S5.

### Adjusted association of BP with various components of body composition

As shown in Table 2, stepwise multiple linear regression analyses showed that, after adjustment for covariates, SBP were significantly determined by trunk fat mass (β = 0.33, *P* < 0.001), leg fat mass (β = −0.12, *P* < 0.001), and total muscle mass (β = 0.10, *P* < 0.001), and DBP were significantly determined by trunk fat mass (β = 0.52, *P* < 0.001), leg fat mass (β = −0.15, *P* < 0.001), arm fat mass (β = −0.23, *P* < 0.001), and total muscle mass (β = 0.06, *P* < 0.001) in men. As to women, after adjusted for covariates, SBP were significantly determined by trunk fat mass (β = 0.25, *P* < 0.001), leg fat mass (β = −0.12, *P* < 0.001), and total muscle mass (β = 0.08, *P* < 0.001), and DBP were significantly determined by trunk fat mass (β = 0.34, *P* < 0.001), leg fat mass (β = −0.06, P=0.003), and arm fat mass (β = −0.12, *P* < 0.001), as seen in Table 3. Further adjustment for BMI observed similar results in both men and women.

**Table 2:** Impact of various components of body composition on BP in men.

**Models**	**SBP**				**DBP**			
	**β**	**SE**	***P***	**R^2^**	**β**	**SE**	***P***	**R^2^**
**Model 1**				0.074				0.116
Trunk fat mass	0.33	0.05	<0.001		0.70	0.06	<0.001	
Leg fat mass	−0.22	0.08	<0.001		−0.28	0.08	<0.001	
Arm fat mass					−0.25	0.34	<0.001	
Total muscle mass	0.13	0.02	<0.001		0.06	0.02	<0.001	
**Model 2**				0.142				0.200
Trunk fat mass	0.33	0.08	<0.001		0.52	0.07	<0.001	
Leg fat mass	−0.12	0.11	<0.001		−0.15	0.09	<0.001	
Arm fat mass	−0.07	0.39	0.064		−0.23	0.33	<0.001	
Total muscle mass	0.10	0.02	<0.001		0.06	0.02	<0.001	
**Model 3**				0.148				0.200
Trunk fat mass	0.25	0.09	<0.001		0.50	0.07	<0.001	
Leg fat mass	−0.12	0.11	<0.001		−0.15	0.09	<0.001	
Arm fat mass	−0.12	0.41	0.002		−0.24	0.34	<0.001	
Total muscle mass	0.04	0.03	<0.001		0.05	0.02	0.006	

Multiple stepwise regression analyses were performed. Model 1 included trunk fat mass, leg fat mass, arm fat mass and total muscle mass. Model 2 further adjusted for potential covariates including age, race/ethnicity, education level, physical activity, diabetes mellitus, low-density lipoprotein cholesterol, total cholesterol, anti-diabetes medication and lipid-lowering medication. Model 3 further adjusted for body mass index on the basis of Model 2.

SBP, systolic blood pressure; DBP, diastolic blood pressure; SE, standard error. β indicates standardized regression coefficient.

**Table 3:** Impact of different components of body composition on BP in women.

**Models**	**SBP**				**DBP**			
	**β**	**SE**	***P***	**R^2^**	**β**	**SE**	***P***	**R^2^**
**Model 1**				0.080				0.048
Trunk fat mass	0.38	0.04	<0.001		0.40	0.05	<0.001	
Leg fat mass	−0.14	0.06	<0.001		−0.07	0.05	0.002	
Arm fat mass					−0.12	0.21	<0.001	
Total muscle mass					−0.05	0.02	0.008	
**Model 2**				0.228				0.156
Trunk fat mass	0.25	0.05	<0.001		0.34	0.05	<0.001	
Leg fat mass	−0.12	0.06	<0.001		−0.06	0.05	0.003	
Arm fat mass					−0.12	0.19	<0.001	
Total muscle mass	0.08	0.03	<0.001					
**Model 3**				0.233				0.159
Trunk fat mass	0.11	0.07	<0.001		0.37	0.06	<0.001	
Leg fat mass	−0.17	0.07	<0.001		−0.05	0.05	0.028	
Arm fat mass					−0.11	0.20	<0.001	
Total muscle mass	0.04	0.04	0.053					

Multiple stepwise regression analyses were performed. Model 1 included trunk fat mass, leg fat mass, arm fat mass and total muscle mass. Model 2 further adjusted for potential covariates including age, race/ethnicity, education level, physical activity, diabetes mellitus, low-density lipoprotein cholesterol, total cholesterol, anti-diabetes medication and lipid-lowering medication. Model 3 further adjusted for body mass index on the basis of Model 2.

SBP, systolic blood pressure; DBP, diastolic blood pressure; SE, standard error. β indicates standardized regression coefficient.

In subgroup analyses, the adjusted association of SBP with trunk fat mass and leg fat mass and the adjusted association of DBP with trunk fat mass, leg fat mass and arm fat mass were not altered along with the categories of BMI; however, the adjusted association between SBP and total muscle mass were only observed in overweight and obsess subgroups, but not in normal-weight subgroup, as shown in Supplementary Table S1 for men and Supplementary Table S2 for women. Additionally, stratification by diabetes/dyslipidemia did not alter the adjusted association between BP and the corresponding variables of body composition, as seen in Supplementary Tables S3, S4.

### Proportional contribution of various components of body composition on BP

**Figure 2:**
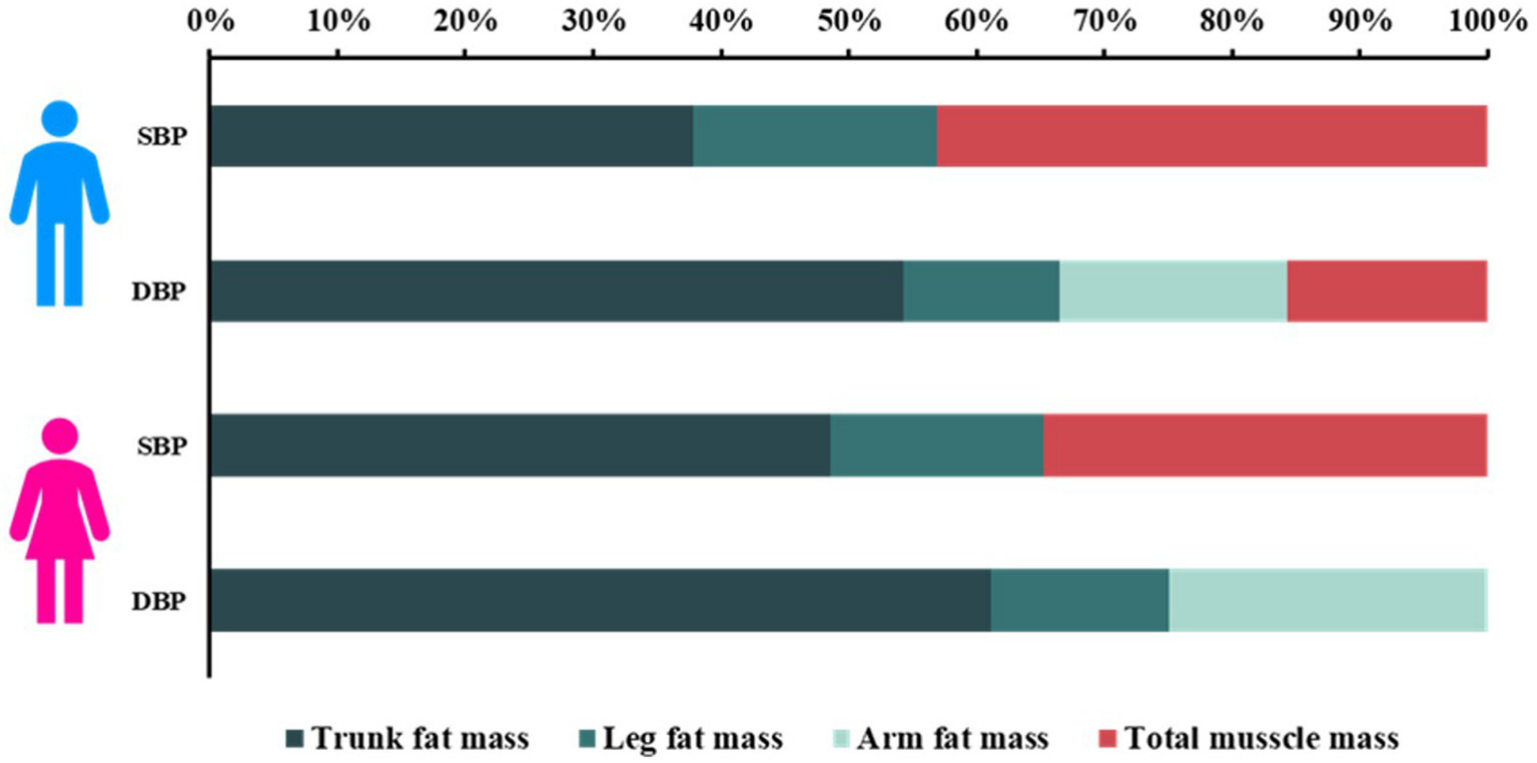
Proportionate contribution of various components of body composition to BP in men and women. SBP, systolic blood pressure; DBP, diastolic blood pressure.

Relative importance analysis indicated that trunk fat mass was the major contributor to SBP and DBP in both men and women; meanwhile, total muscle mass also made relatively great contribution to SBP for men and women, as shown in Figure 2. Leg fat mass contributed relatively less to BP, in contrast to trunk fat mass and total muscle mass. The detailed contribution percentage of each component to BP are presented in Supplementary Table S5.

### Distinct associations of ratios of central vs. peripheral fat/muscle mass with BP

**Figure 3:**
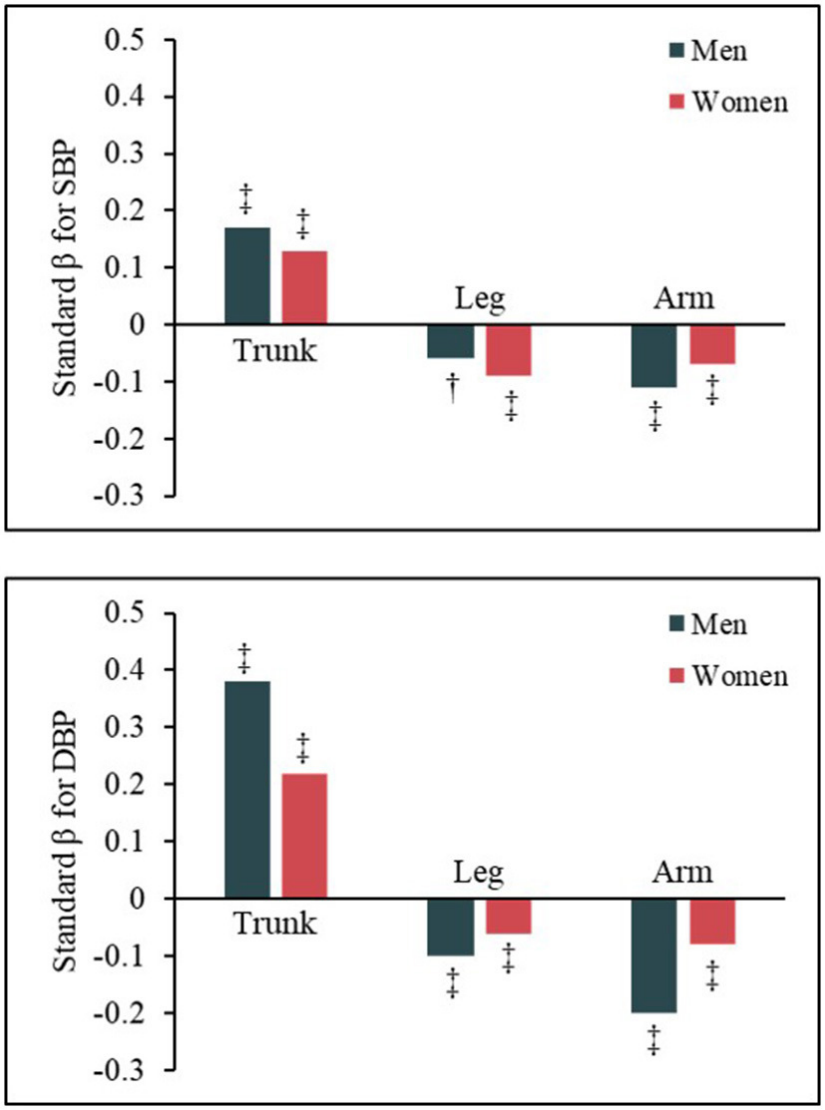
Opposite impact of central and peripheral adiposity on BP.

Multivariable stepwise liner regression model, using ratios of fat and muscle mass in trunk, leg, and arm as independent variables and adjusted for covariates, was also constructed to further determine the impact of central and peripheral adiposity on BP. Results showed that trunk FMR (indicating central adiposity) was significantly and positively associated with SBP (β = 0.17 for men; β = 0.13 for women; *P* < 0.001 for both sexes) and DBP (β = 0.38 for men; β = 0.22 for women; *P* < 0.001 for both sexes); however, leg FMR and arm FMR (both indicates peripheral adiposity) were significantly and negatively associated with SBP (Leg: β = −0.06 for men; β = −0.09 for women; *P* < 0.005 for both sexes; Arm: β = −0.11 for men; β = −0.07 for women; *P* < 0.001 for both sexes) and DBP (Leg: β = −0.10 for men; β = −0.06 for women; *P* < 0.001 for both sexes; Arm: β = −0.20 for men; β = −0.08 for women; *P* < 0.001 for both sexes), in men and women (as shown in Figure 3 and in Supplementary Tables S6, S7 for more details).

### Addition of TLR on BMI in determining BP

For each tertile of body mass index, SBP significantly increased along with TLR increasing from the lowest to the highest tertile, in both men and women (P for trend < 0.001 for both sexes, as shown in Supplementary Figure S6). Similar trends were also found for DBP in both sexes.

## Discussion

In the present study, we comprehensively investigated the association of BP with body composition and fat distribution in a large cohort with young and middle-aged participants. Our results indicated that both fat mass and muscle mass were independently associated with and substantially contributed to SBP in both men and women. Furthermore, the association of fat mass with BP varies along with fat distribution. After adjustment for other components of body composition and potential clinical covariates, trunk fat mass was positively associated with both SBP and DBP, and was the most dominant contributor to BP; however, leg fat mass was negatively associated with both SBP and DBP.

By determining the basic metabolism of human body, body size and composition significantly associates with BP and hypertension (25). Whole-body fat mass, as one of the important components of body composition, has been extensively studied in the past. However, it might be time to breakdown total fat mass into parts, given that adipose tissues in various depots are presented with distinct biological effects on human metabolism. Epidemiological data even showed that trunk and limb fat have opposite effects on BP (13–17). However, it should be noted that these previous studies rarely adjusted for total lean/muscle mass. In our study, we investigated a total of 14,412 participants from the 1999–2018 NHANES and adjusted for other depots of adiposity, muscle mass and clinical variates. We confirmed the independently positive association of trunk fat mass and the independently negative association of leg fat mass with BP. Furthermore, we firstly revealed that trunk fat mass contributed most (38–61%) to BP, followed by total muscle mass (16–43%) and leg/arm fat mass (12–25%). Of note, although adiposity in both upper and lower limbs negatively effects BP, it seems that adiposity in lower limbs has more impact on SBP, in contrast to adiposity in upper limbs. Although the tight link between leg fat and BP indicated by these cross-sectional studies, however, no independent association were found between the incidence of hypertension and lower body fat mass after adjusted for abdominal visceral and subcutaneous adiposities in a longitudinal study with 7-year follow up by Chandra and colleagues (26). Accordingly, Chandra et al. thought that lower body fat might have a less important role in preventing hypertension. Truly, relative importance analysis in our study showed that leg fat mass contributed less to BP, compared with trunk fat and muscle mass. However, more investigations, especially longitudinal studies, are warranted to further confirm these observations.

Considering the opposite effects of trunk and leg fat mass on human metabolism, the ratio between trunk and leg fat mass, as a combined indicator of trunk fat and leg fat, may be a more useful index for evaluating and predicting cardiometabolic risk. As indicated by Zhang et al., an increased leg/trunk fat mass ratio strongly and independently associated with lower levels of most risk factors and decreased odds of metabolic syndrome (15). In addition, the ratios between trunk and leg fat mass were also reported to be significantly associated with cardiovascular risk factors and diseases (27–29). Further efforts are warranted to confirmed the potential superiority of the ratio indices, based on trunk and leg fat mass, to predict cardiometabolic profile and risk.

It is obvious that researches on body composition have been unfairly focused on fat mass, due to the prevailing of overweigh/obesity and the increasing rate of adipose accumulation along with aging (30). However, epidemiological data also have demonstrated that body lean mass significantly associates with cardiometabolic risk and mortality (31, 32). As to the impact of total lean/muscle mass on BP, inconsistent results were obtained. Several early studies focusing on the percentage of total lean/muscle mass reported a negative correlation between total lean/muscle mass and BP (12). The velocity of adipose increase is faster than the increase of lean/muscle mass, which leads to a relatively decrease of percentage of lean/muscle mass with aging. Analysis based on the percentage of total lean/muscle mass would inevitably cause a negative association between total lean/muscle mass and BP. Theoretically, there should be a positive correlation between body muscle mass and BP, due to the increased cardiac output (or blood volume) driven by raised metabolic need related to increased body muscle mass (21, 33, 34). Indeed, positive associations between BP and mass of body muscle were observed in many studies (11, 20, 21, 35, 36). Importantly, the effect of body fat distribution was not taken into account in these previous studies. In our present study, we simultaneously examined the effects of body muscle mass and body fat mass in various depots on BP and confirmed a significantly positive association between body muscle mass and BP in both sexes. Furthermore, for the first time, we indicated that body muscle mass was also a major contributor to SBP, just following the contribution by trunk fat mass, in both sexes.

Some researchers suggest that fat mass represents metabolic load and muscle mass represents metabolic capacity, and that they interact to determine metabolic risk (24). Therefore, FMR, combined fat mass and muscle mass, has been suggested as a novel indictor for assessment obesity. Whole-body FMR has been shown to be significantly associated with hypertension (37), metabolic syndrome (38) and type 2 diabetes (39). In our study, we found that FMR in different body regions showed different relationship with BP, as did fat mass in different regions. Wang et al. reported all FMR in different regions showed an increase risk of type 2 diabetes (39). However, the biggest limitation of this study is that they did not take into consideration that different body fat distribution has different effects on glucose metabolism.

The data presented in the present study should be interpreted under their limitations. First, due to the cross-sectional nature of this study, we were not able to infer causality between body composition and BP elevation. Second, hypertensive patients taking BP-lowering drugs were excluded from this study, which may add some uncertainty to our study. Third, considering the scope of study participants, the findings drawn from our study may not be applicable to the elderly and patients with cardiovascular diseases. Fourth, trunk adiposity contains two types of adipose tissue, namely visceral and subcutaneous adipose tissue which have different characteristics and biological function. In our analysis, it was not possible for us to further breakdown trunk fat mass into the two types of adipose tissue. Nevertheless, we provided a quite comprehensive investigation and understanding on the association between the weight of components of body composition and BP. Fifth, it should be noted that dietary factors such as alcohol drinking (40) and total calorie intake (41) have important impacts on BP, however, we were not able to take these factors into account in our models. Future studies may further explore the influence of dietary factors on the associations of BP with body composition and fat distribution.

## Conclusion

To conclude, our study confirmed the tight link between body composition and BP, indicating that both fat mass and muscle mass were independently associated with and substantially contributed to BP (especially SBP). Furthermore, trunk fat mass was positively associated with BP and was the most dominant contributor to BP, whereas leg fat mass was negatively associated with BP, after adjustment for other components of body composition and potential covariates. These findings suggest that the impact of lean/muscle mass on BP should not be neglected. Future investigations are needed to confirm the biological and clinical differences of trunk and leg adipose tissue and the corresponding mechanisms underlying these differences.

## Data availability statement

The datasets presented in this study can be found in online repositories. The names of the repository/repositories and accession number(s) can be found below: https://www.cdc.gov/nchs/nhanes/index.htm.

## Ethics statement

The studies involving human participants were reviewed and approved by National Center for Health Statistics Research Ethics Review Board. The patients/participants provided their written informed consent to participate in this study.

## Author contributions

SZ curated data, performed literature search and the analysis, interpreted the results, and drafted the manuscript. SY conceived the idea, curated data, formulated and performed data analysis, interpreted the results, drafted, and revised the manuscript. JT, YZhao, and CX interpreted the data and reviewed the manuscript. YX and YZhan reviewed and revised the manuscript. All authors contributed to the article and approved the submitted version.

## Funding

SY was supported by Talent Program of Shanghai Tenth People's Hospital Affiliated to Tongji University (2021SYPDRC043). YZhao was supported by Clinical Research Project of Shanghai Municipal Health Commission (No. 20214Y0152). YZhan was supported by National Nature Science Foundation of China (82170388), Clinical Research Plan of SHDC (No. SHDC2020CR1040B), Shanghai Technology Research Leader Program (21XD1434700), Shanghai Three-year Plan for Biobank Construction Project (SHDC2020CR5009, SHDC2020CR5015-002), and the Cardiac Rehabilitation Fund by the International Medical Exchange Foundation (Z-2019-42-1908-3).

## Conflict of interest

The authors declare that the research was conducted in the absence of any commercial or financial relationships that could be construed as a potential conflict of interest.

## Publisher's note

All claims expressed in this article are solely those of the authors and do not necessarily represent those of their affiliated organizations, or those of the publisher, the editors and the reviewers. Any product that may be evaluated in this article, or claim that may be made by its manufacturer, is not guaranteed or endorsed by the publisher.
