# Supplementary material for: The impact of body composition and fat distribution on blood pressure in young and middle-aged adults
Source: Front Nutr. 2022 Sep 2;9:979042. doi: 10.3389/fnut.2022.979042 (PMC9478411; doi:10.3389/fnut.2022.979042)
Supplement: Supplementary file 1 [file Data_Sheet_1.docx]

**Supplementary files**

**The Impact of Body Composition and Fat Distribution on Blood Pressure in Young and Middle-aged Adults**

**Running title:** Impact of Body Composition on BP

Song Zhao, Jiamin Tang, Yifan Zhao, Chong Xu, Yawei Xu, Shikai Yu^#^, Yi Zhang^#^

Department of Cardiology, Shanghai Tenth People’s Hospital, Tongji University School of Medicine

^#^Indicate correspondence authors

**Correspondence:**

Prof. Yi Zhang

Department of Cardiology, Shanghai Tenth People’s Hospital, Tongji University School of Medicine

Email: yizshcn@gmail.com

Dr. Shikai Yu

Department of Cardiology, Shanghai Tenth People’s Hospital, Tongji University School of Medicine

Email: shikaiyu@yahoo.com

**Table S1. Association between BP and body composition in subgroups stratified by BMI in men**

| **Subgroups/Models** | **SBP** | | | | **DBP** | | | |
| --- | --- | --- | --- | --- | --- | --- | --- | --- |
|  | **β** | **SE** | **P** | **R^2^** | **β** | **SE** | **P** | **R^2^** |
| **18.5≤BMI<25 (n=2526)** |  |  |  |  |  |  |  |  |
| **Model 1** |  |  |  | 0.018 |  |  |  | 0.069 |
| Trunk fat mass | 0.17 | 0.14 | <0.001 |  | 0.35 | 0.12 | <0.001 |  |
| Leg fat mass | -0.13 | 0.22 | <0.001 |  | -0.21 | 0.18 | <0.001 |  |
| Arm fat mass |  |  |  |  |  |  |  |  |
| Total muscle mass | 0.04 | 0.04 | 0.036 |  |  |  |  |  |
| **Model 2** |  |  |  | 0.118 |  |  |  | 0.172 |
| Trunk fat mass | 0.20 | 0.21 | <0.001 |  | 0.23 | 0.18 | <0.001 |  |
| Leg fat mass | -0.13 | 1.01 | <0.001 |  | -0.07 | 0.23 | 0.048 |  |
| Arm fat mass |  |  |  |  | -0.11 | 1.06 | 0.011 |  |
| Total muscle mass |  |  |  |  |  |  |  |  |
| **25≤BMI<30 (n=2793)** |  |  |  |  |  |  |  |  |
| **Model 1** |  |  |  | 0.030 |  |  |  | 0.099 |
| Trunk fat mass | 0.14 | 0.10 | <0.001 |  | 0.38 | 0.11 | <0.001 |  |
| Leg fat mass | -0.18 | 0.15 | <0.001 |  | -0.21 | 0.15 | <0.001 |  |
| Arm fat mass |  |  |  |  | -0.08 | 0.68 | 0.014 |  |
| Total muscle mass | 0.08 | 0.04 | <0.001 |  | 0.09 | 0.03 | <0.001 |  |
| **Model 2** |  |  |  | 0.103 |  |  |  | 0.183 |
| Trunk fat mass | 0.13 | 0.12 | <0.001 |  | 0.29 | 0.11 | <0.001 |  |
| Leg fat mass | -0.14 | 0.17 | <0.001 |  | -0.11 | 0.16 | <0.001 |  |
| Arm fat mass |  |  |  |  | -0.10 | 0.66 | 0.003 |  |
| Total muscle mass | 0.07 | 0.04 | <0.001 |  | 0.08 | 0.03 | <0.001 |  |
| **BMI≥30 (n=1870)** |  |  |  |  |  |  |  |  |
| **Model 1** |  |  |  | 0.046 |  |  |  | 0.082 |
| Trunk fat mass | 0.27 | 0.11 | <0.001 |  | 0.51 | 0.10 | <0.001 |  |
| Leg fat mass | -0.13 | 0.14 | <0.001 |  | -0.22 | 0.13 | <0.001 |  |
| Arm fat mass | -0.09 | 0.52 | 0.081 |  | -0.27 | 0.46 | <0.001 |  |
| Total muscle mass | 0.12 | 0.04 | <0.001 |  | 0.06 | 0.04 | 0.035 |  |
| **Model 2** |  |  |  | 0.098 |  |  |  | 0.163 |
| Trunk fat mass | 0.29 | 0.13 | <0.001 |  | 0.47 | 0.11 | <0.001 |  |
| Leg fat mass | -0.09 | 0.16 | 0.038 |  | -0.13 | 0.14 | 0.001 |  |
| Arm fat mass | -0.10 | 0.52 | 0.041 |  | -0.25 | 0.48 | <0.001 |  |
| Total muscle mass | 0.09 | 0.04 | 0.003 |  |  |  |  |  |

Multiple stepwise regression analyses were performed. Model 1 included trunk fat mass, leg fat mass, arm fat mass and total muscle mass. Model 2 further adjusted for potential covariates including age, race/ethnicity, education level, physical activity, diabetes mellitus, low-density lipoprotein cholesterol, total cholesterol, anti-diabetes medication and lipid-lowering medication. BMI, body mass index; BP, blood pressure; SBP, systolic blood pressure; DBP, diastolic blood pressure; SE, standard error. β indicates standardized regression coefficient.

**Table S2. Association between BP and body composition in subgroups stratified by BMI in women**

| **Subgroups/Models** | **SBP** | | | | **DBP** | | | |
| --- | --- | --- | --- | --- | --- | --- | --- | --- |
|  | **β** | **SE** | **P** | **R^2^** | **β** | **SE** | **P** | **R^2^** |
| **18.5≤BMI<25 (N=2620)** |  |  |  |  |  |  |  |  |
| **Model 1** |  |  |  | 0.043 |  |  |  | 0.036 |
| Trunk fat mass | 0.23 | 0.12 | <0.001 |  | 0.21 | 0.09 | <0.001 |  |
| Leg fat mass | -0.11 | 0.15 | <0.001 |  | -0.07 | 0.11 | <0.001 |  |
| Arm fat mass |  |  |  |  |  |  |  |  |
| Total muscle mass |  |  |  |  |  |  |  |  |
| **Model 2** |  |  |  | 0.196 |  |  |  | 0.161 |
| Trunk fat mass | 0.14 | 0.13 | <0.001 |  | 0.13 | 0.10 | <0.001 |  |
| Leg fat mass | -0.07 | 0.15 | <0.001 |  | -0.05 | 0.11 | 0.019 |  |
| Arm fat mass |  |  |  |  |  |  |  |  |
| Total muscle mass |  |  |  |  |  |  |  |  |
| **25≤BMI<30 (n=1975)** |  |  |  |  |  |  |  |  |
| **Model 1** |  |  |  | 0.035 |  |  |  | 0.036 |
| Trunk fat mass | 0.12 | 0.14 | <0.001 |  | 0.22 | 0.12 | <0.001 |  |
| Leg fat mass | -0.18 | 0.14 | <0.001 |  | -0.08 | 0.11 | 0.003 |  |
| Arm fat mass |  |  |  |  | -0.08 | 0.49 | 0.014 |  |
| Total muscle mass |  |  |  |  |  |  |  |  |
| **Model 2** |  |  |  | 0.195 |  |  |  | 0.151 |
| Trunk fat mass | 0.05 | 0.14 | 0.011 |  | 0.19 | 0.12 | <0.001 |  |
| Leg fat mass | -0.15 | 0.14 | <0.001 |  | -0.08 | 0.11 | 0.003 |  |
| Arm fat mass |  |  |  |  | -0.09 | 0.47 | 0.002 |  |
| Total muscle mass | 0.06 | 0.07 | 0.08 |  |  |  |  |  |
| **BMI≥30 (n=2320)** |  |  |  |  |  |  |  |  |
| **Model 1** |  |  |  | 0.025 |  |  |  | 0.022 |
| Trunk fat mass | 0.14 | 0.10 | <0.001 |  | 0.23 | 0.07 | <0.001 |  |
| Leg fat mass | -0.08 | 0.09 | 0.002 |  | -0.08 | 0.25 | 0.025 |  |
| Arm fat mass | 0.06 | 0.35 | 0.083 |  |  |  |  |  |
| Total muscle mass |  |  |  |  | -0.06 | 0.04 | 0.048 |  |
| **Model 2** |  |  |  | 0.197 |  |  |  | 0.129 |
| Trunk fat mass | 0.14 | 0.08 | <0.001 |  | 0.21 | 0.07 | <0.001 |  |
| Leg fat mass | -0.08 | 0.09 | 0.002 |  | -0.09 | 0.23 | 0.005 |  |
| Arm fat mass |  |  |  |  |  |  |  |  |
| Total muscle mass | 0.10 | 0.05 | <0.001 |  |  |  |  |  |

Multiple stepwise regression analyses were performed. Model 1 included trunk fat mass, leg fat mass, arm fat mass and total muscle mass. Model 2 further adjusted for potential covariates including age, race/ethnicity, education level, physical activity, diabetes mellitus, low-density lipoprotein cholesterol, total cholesterol, anti-diabetes medication and lipid-lowering medication. BMI, body mass index, BP, blood pressure; SBP, systolic blood pressure; DBP, diastolic blood pressure; SE, standard error. β indicates standardized regression coefficient.

**Table S3. Association between BP and body composition stratified by dyslipidemia and diabetes in men**

| **Subgroups/Models** | **SBP** | | | | **DBP** | | | |
| --- | --- | --- | --- | --- | --- | --- | --- | --- |
|  | **β** | **SE** | **P** | **R^2^** | **β** | **SE** | **P** | **R^2^** |
| **Without dyslipidemia or diabetes (n=2482)** | | | | | | | | |
| **Model 1** |  |  |  | 0.068 |  |  |  | 0.086 |
| Trunk fat mass | 0.31 | 0.10 | <0.001 |  | 0.62 | 0.13 | <0.001 |  |
| Leg fat mass | -0.22 | 0.16 | <0.001 |  | -0.33 | 0.16 | <0.001 |  |
| Arm fat mass |  |  |  |  | -0.18 | 0.72 | 0.017 |  |
| Total muscle mass | 0.15 | 0.04 | <0.001 |  | 0.09 | 0.03 | <0.001 |  |
| **Model 2** |  |  |  | 0.149 |  |  |  | 0.176 |
| Trunk fat mass | 0.28 | 0.12 | <0.001 |  | 0.43 | 0.14 | <0.001 |  |
| Leg fat mass | -0.14 | 0.17 | <0.001 |  | -0.21 | 0.16 | <0.001 |  |
| Arm fat mass |  |  |  |  | -0.13 | 0.69 | 0.074 |  |
| Total muscle mass | 0.10 | 0.04 | <0.001 |  | 0.06 | 0.03 | 0.027 |  |
| **With dyslipidemia and/or diabetes (n=4831)** | | | | | | | | |
| **Model 1** |  |  |  | 0.063 |  |  |  | 0.096 |
| Trunk fat mass | 0.31 | 0.06 | <0.001 |  | 0.64 | 0.07 | <0.001 |  |
| Leg fat mass | -0.21 | 0.10 | <0.001 |  | -0.25 | 0.10 | <0.001 |  |
| Arm fat mass |  |  |  |  | -0.26 | 0.39 | <0.001 |  |
| Total muscle mass | 0.12 | 0.03 | <0.001 |  | 0.05 | 0.02 | 0.007 |  |
| **Model 2** |  |  |  | 0.131 |  |  |  | 0.180 |
| Trunk fat mass | 0.29 | 0.07 | <0.001 |  | 0.53 | 0.08 | <0.001 |  |
| Leg fat mass | -0.15 | 0.11 | <0.001 |  | -0.13 | 0.11 | <0.001 |  |
| Arm fat mass |  |  |  |  | -0.30 | 0.37 | <0.001 |  |
| Total muscle mass | 0.09 | 0.03 | <0.001 |  | 0.06 | 0.02 | 0.004 |  |

Multiple stepwise regression analyses were performed. Model 1 included trunk fat mass, leg fat mass, arm fat mass and total muscle mass. Model 2 further adjusted for potential covariates including age, race/ethnicity, education level, physical activity, diabetes mellitus, low-density lipoprotein cholesterol, total cholesterol, anti-diabetes medication and lipid-lowering medication. SBP, systolic blood pressure; DBP, diastolic blood pressure; SE, standard error. β indicates standardized regression coefficient.

**Table S4. Association between BP and body composition stratified by dyslipidemia and diabetes in women**

| **Subgroups/Models** | **SBP** | | | | **DBP** | | | |
| --- | --- | --- | --- | --- | --- | --- | --- | --- |
|  | **β** | **SE** | **P** | **R^2^** | **β** | **SE** | **P** | **R^2^** |
| **Without dyslipidemia or diabetes (n=2460)** | | | | | | | | |
| **Model 1** |  |  |  | 0.076 |  |  |  | 0.032 |
| Trunk fat mass | 0.29 | 0.09 | <0.001 |  | 0.32 | 0.10 | <0.001 |  |
| Leg fat mass | -0.14 | 0.12 | <0.001 |  | -0.16 | 0.38 | 0.003 |  |
| Arm fat mass |  |  |  |  |  |  |  |  |
| Total muscle mass | 0.13 | 0.05 | <0.001 |  |  |  |  |  |
| **Model 2** |  |  |  | 0.176 |  |  |  | 0.131 |
| Trunk fat mass | 0.24 | 0.09 | <0.001 |  | 0.26 | 0.10 | <0.001 |  |
| Leg fat mass | -0.14 | 0.12 | <0.001 |  | -0.14 | 0.37 | 0.004 |  |
| Arm fat mass |  |  |  |  |  |  |  |  |
| Total muscle mass | 0.11 | 0.05 | <0.001 |  |  |  |  |  |
| **With dyslipidemia and/or diabetes (n=4639)** | | | | | | | | |
| **Model 1** |  |  |  | 0.060 |  |  |  | 0.037 |
| Trunk fat mass | 0.34 | 0.05 | <0.001 |  | 0.37 | 0.06 | <0.001 |  |
| Leg fat mass | -0.15 | 0.08 | <0.001 |  | -0.07 | 0.06 | 0.0043 |  |
| Arm fat mass |  |  |  |  | -0.11 | 0.24 | 0.007 |  |
| Total muscle mass |  |  |  |  | -0.06 | 0.03 | 0.012 |  |
| **Model 2** |  |  |  | 0.234 |  |  |  | 0.155 |
| Trunk fat mass | 0.25 | 0.06 | <0.001 |  | 0.34 | 0.06 | <0.001 |  |
| Leg fat mass | -0.11 | 0.08 | <0.001 |  | -0.08 | 0.06 | 0.002 |  |
| Arm fat mass |  |  |  |  | -0.12 | 0.22 | 0.002 |  |
| Total muscle mass | 0.06 | 0.04 | 0.005 |  |  |  |  |  |

Multiple stepwise regression analyses were performed. Model 1 included trunk fat mass, leg fat mass, arm fat mass and total muscle mass. Model 2 further adjusted for potential covariates including age, race/ethnicity, education level, physical activity, diabetes mellitus, low-density lipoprotein cholesterol, total cholesterol, anti-diabetes medication and lipid-lowering medication. SBP, systolic blood pressure; DBP, diastolic blood pressure; SE, standard error. β indicates standardized regression coefficient.

**Table S5. The proportional contribution of various components of body composition to BP**

|  | Trunk fat mass | Leg fat mass | Arm fat mass | Total muscle mass |
| --- | --- | --- | --- | --- |
| Men |  |  |  |  |
| SBP | 37.9% | 19.1% | - | 43.1% |
| DBP | 54.3% | 12.3% | 17.8% | 15.7% |
| Women |  |  |  |  |
| SBP | 48.6% | 16.7% | - | 34.7% |
| DBP | 61.1% | 14.0% | 24.9% | - |

Variables with statistical significance in multiple stepwise regression (model 2) were included in relative importance analysis and the excluded variables were indicated as -. SBP, systolic blood pressure; DBP, diastolic blood pressure;

**Table S6. Association of BP with FMR of various body parts in men**

| **Models** | **SBP** | | | | **DBP** | | | |
| --- | --- | --- | --- | --- | --- | --- | --- | --- |
|  | **β** | **SE** | **P** | **R^2^** | **β** | **SE** | **P** | **R^2^** |
| **Model 1** |  |  |  | 0.056 |  |  |  | 0.112 |
| Trunk FMR | 0.41 | 2.27 | <0.001 |  | 0.62 | 1.90 | <0.001 |  |
| Leg FMR | -0.12 | 2.22 | <0.001 |  | -0.20 | 1.86 | <0.001 |  |
| Arm FMR | -0.12 | 3.10 | <0.001 |  | -0.23 | 2.59 | <0.001 |  |
| **Model 2** |  |  |  | 0.128 |  |  |  | 0.195 |
| Trunk FMR | 0.32 | 2.44 | <0.001 |  | 0.45 | 2.02 | <0.001 |  |
| Leg FMR | -0.07 | 2.24 | 0.001 |  | -0.11 | 1.86 | <0.001 |  |
| Arm FMR | -0.08 | 3.03 | 0.007 |  | -0.19 | 2.51 | <0.001 |  |
| **Model 3** |  |  |  | 0.145 |  |  |  | 0.199 |
| Trunk FMR | 0.17 | 2.70 | <0.001 |  | 0.38 | 2.26 | <0.001 |  |
| Leg FMR | -0.06 | 2.22 | 0.004 |  | -0.10 | 1.86 | <0.001 |  |
| Arm FMR | -0.11 | 3.02 | <0.001 |  | -0.20 | 2.52 | <0.001 |  |

Multiple stepwise regression analyses were performed. Model 1 included trunk fat mass, leg fat mass, arm fat mass and total muscle mass. Model 2 further adjusted for potential covariates including age, race/ethnicity, education level, physical activity, diabetes mellitus, low-density lipoprotein cholesterol, total cholesterol, anti-diabetes medication and lipid-lowering medication. Model 3 further adjusted for body mass index on the basis of Model 2. SBP, systolic blood pressure; DBP, diastolic blood pressure; SE, standard error. β indicates standardized regression coefficient.

**Table S7. Association of BP with FMR of various body parts in women**

| **Models** | **SBP** | | | | **DBP** | | | |
| --- | --- | --- | --- | --- | --- | --- | --- | --- |
|  | **β** | **SE** | **P** | **R^2^** | **β** | **SE** | **P** | **R^2^** |
| **Model 1** |  |  |  | 0.075 |  |  |  | 0.046 |
| Trunk FMR | 0.37 | 1.50 | <0.001 |  | 0.29 | 1.06 | <0.001 |  |
| Leg FMR | -0.10 | 1.21 | <0.001 |  | -0.05 | 0.86 | 0.002 |  |
| Arm FMR | -0.06 | 1.33 | 0.019 |  | -0.06 | 0.94 | 0.014 |  |
| **Model 2** |  |  |  | 0.220 |  |  |  | 0.156 |
| Trunk FMR | 0.25 | 1.03 | <0.001 |  | 0.26 | 1.07 | <0.001 |  |
| Leg FMR | -0.11 | 1.00 | <0.001 |  | -0.06 | 0.83 | <0.001 |  |
| Arm FMR |  |  |  |  | -0.07 | 0.91 | 0.004 |  |
| **Model 3** |  |  |  | 0.233 |  |  |  | 0.156 |
| Trunk FMR | 0.13 | 1.71 | <0.001 |  | 0.22 | 1.25 | <0.001 |  |
| Leg FMR | -0.09 | 1.13 | <0.001 |  | -0.06 | 0.83 | <0.001 |  |
| Arm FMR | -0.07 | 1.27 | <0.001 |  | -0.08 | 0.93 | <0.001 |  |

Multiple stepwise regression analyses were performed. Model 1 included trunk fat mass, leg fat mass, arm fat mass and total muscle mass. Model 2 further adjusted for potential covariates including age, race/ethnicity, education level, physical activity, diabetes mellitus, low-density lipoprotein cholesterol, total cholesterol, anti-diabetes medication and lipid-lowering medication. Model 3 further adjusted for body mass index on the basis of Model 2. SBP, systolic blood pressure; DBP, diastolic blood pressure; SE, standard error. β indicates standardized regression coefficient.

**Figure S1. Difference in body composition between men and women**

**
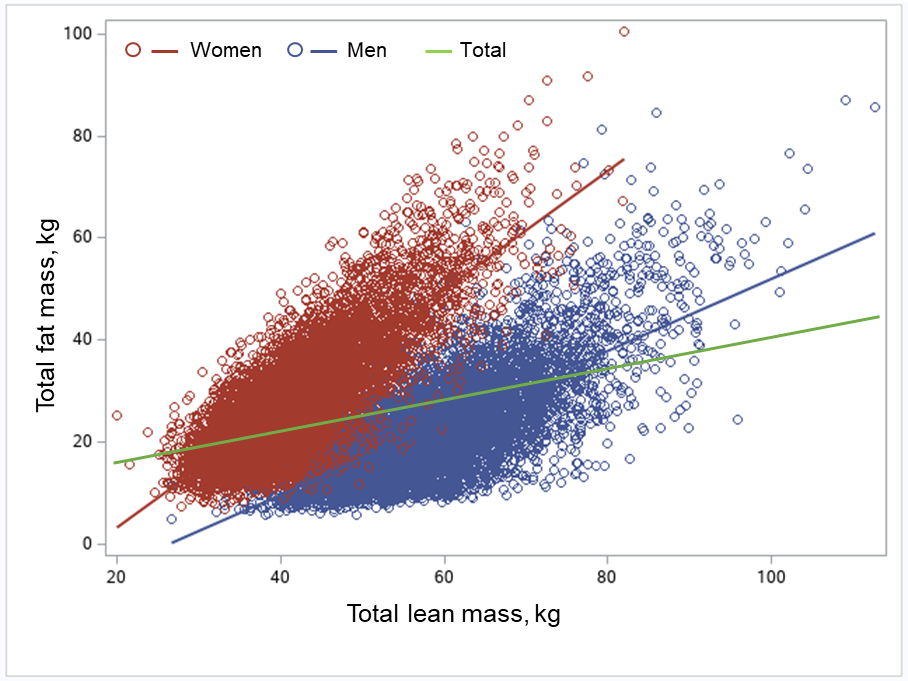
**

Correlation coefficient in male=0.71; Correlation coefficient in female=0.80; Correlation coefficient in total population=0.34.

**Figure S2. Correlation between various components of body composition in men**

**Figure S3. Correlation between various components of body composition in women**

**Figure S4. Association of BP with residuals from the regression of TMM on TFM in men (upper) and women (lower)**


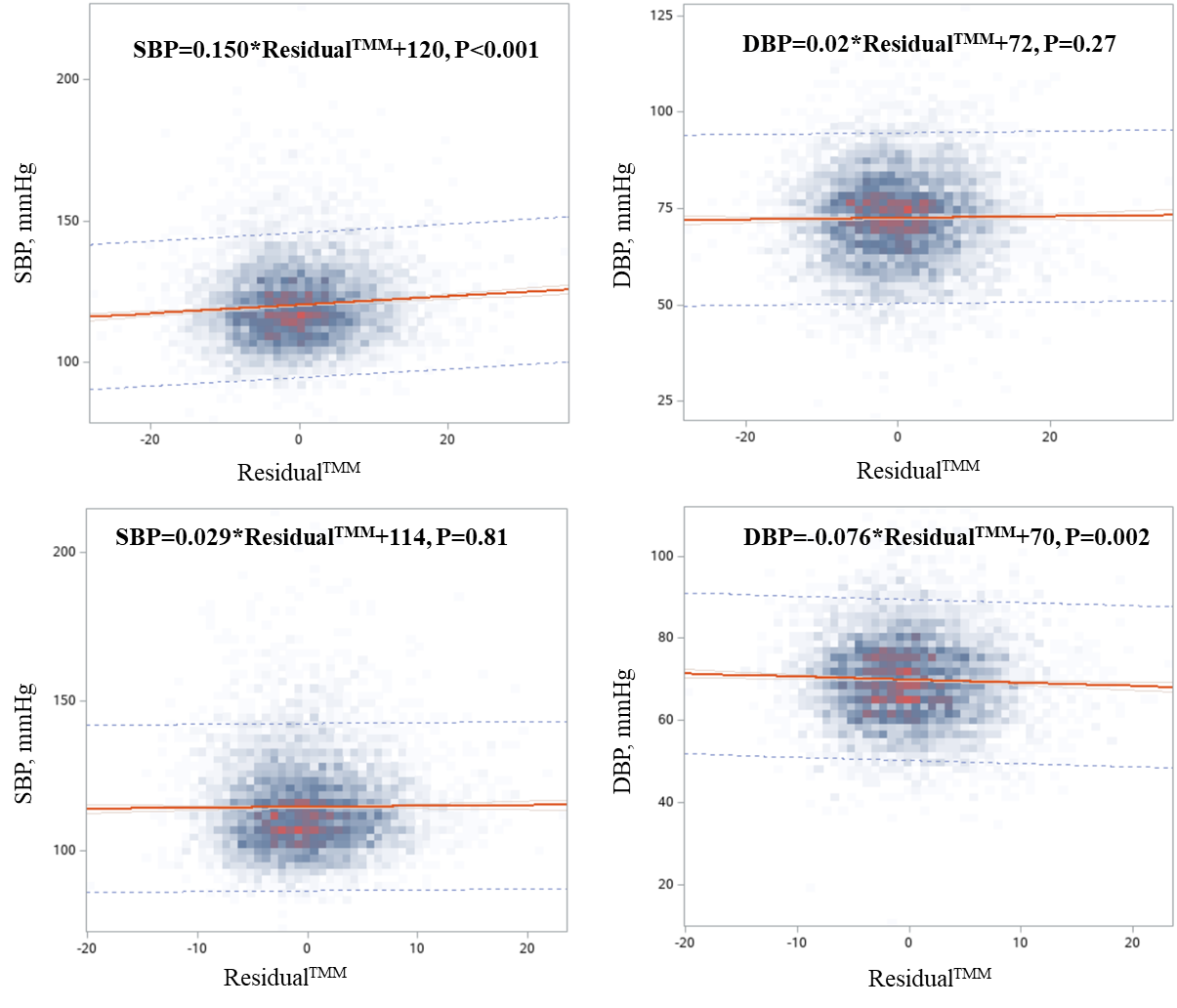


SBP, systolic blood pressure; DBP, systolic blood pressure; TFM, total fat mass; TMM, total muscle mass.

**Figure S5. Association of BP with residuals from the regression of LFM on TrFM in men (upper) and women (lower)**


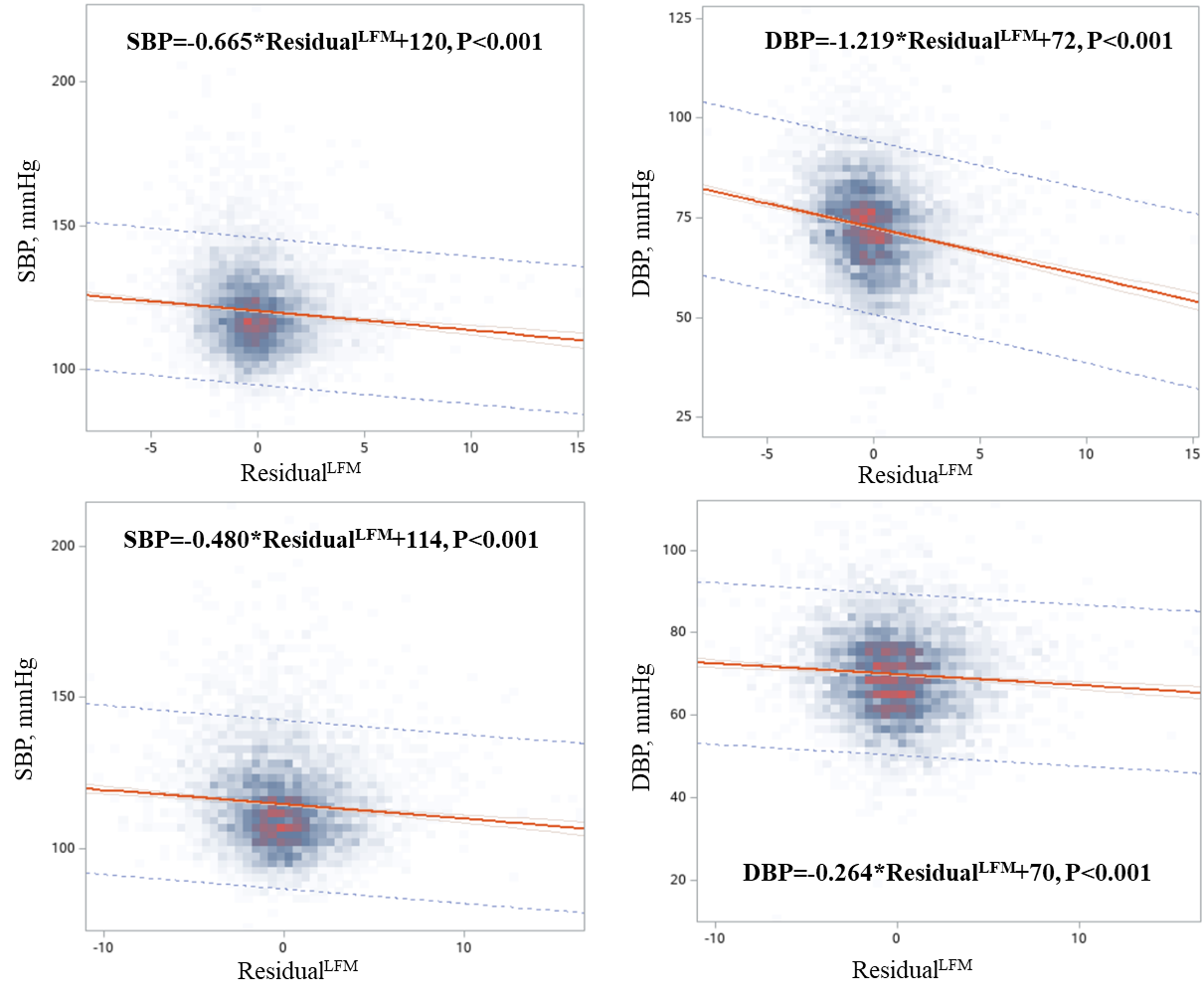


SBP, systolic blood pressure; DBP, diastolic blood pressure; TrFM, trunk fat mass; LFM, leg fat mass.

**Figure S6. Interaction between BMI and TLR in determining BP in men (upper) and women (lower)**


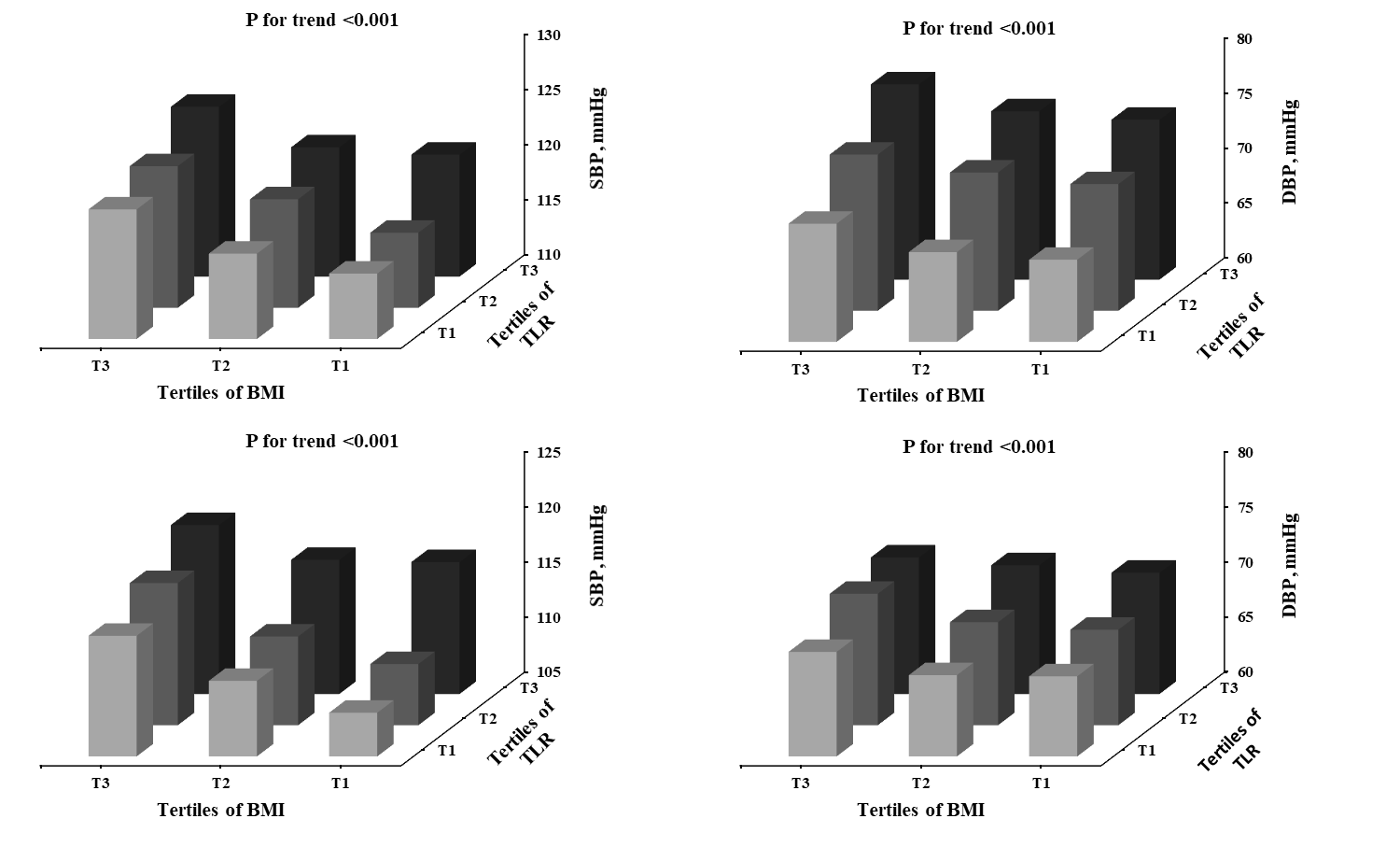
SBP, systolic blood pressure; DBP, diastolic blood pressure; BMI, body mass index; TLR, trunk to leg fat mass ratio.
